# Preparation and Characterization of Poly(acrylic acid-co-vinyl imidazole) Hydrogel-Supported Palladium Catalyst for Tsuji–Trost and Suzuki Reactions in Aqueous Media

**DOI:** 10.3390/gels10120758

**Published:** 2024-11-23

**Authors:** Huijun Song, Amatjan Sawut, Rena Simayi, Yuqi Sun

**Affiliations:** 1 State Key Laboratory of Chemistry and Utilization of Carbon Based Energy Resources, College of Chemistry, Xinjiang University, Urumqi 830017, China; shj1498249640@163.com (H.S.); yuqisun2023@163.com (Y.S.); 2 College of Chemical Engineering, Xinjiang University, Urumqi 830017, China

**Keywords:** heterogeneous catalyst, supported hydrogel Pd catalyst, Tsuji–Trost and Suzuki reactions, repeatable

## Abstract

In this study, a novel polyacrylate-co-vinyl imidazole hydrogel-supported palladium (Pd) catalyst (P(AA-co-VI)@Pd) was prepared through heat-initiated polymerization, starting with the formation of a complex between vinyl imidazole and palladium chloride, followed by the addition of 75% neutralized acrylic acid (AA), crosslinking agent, and initiator. The structure and morphology of the catalyst were characterized using ICP-OES, SEM, EDX, Mapping, FT-IR, TGA, XRD, XPS and TEM techniques. It was confirmed that the catalyst exhibited excellent compatibility with water solvent and uniform distribution of Pd. The P(AA-co-VI)@Pd hydrogel catalyst demonstrated remarkable catalytic activity and ease of separation. Notably, in a Tsuji–Trost reaction, employing water as the solvent, it achieved a conversion rate as high as 94% at very low catalyst dosages, indicating its superior catalytic performance. Moreover, after six consecutive cycles, the catalyst maintained good activity and structural stability, highlighting its exceptional reusability and environmental friendliness. Furthermore, the outstanding efficiency of the catalyst was also observed in a Suzuki coupling reaction where both conversion rate and yield reached 100% and 99%, respectively, within just one hour reaction time, thus further validating its universality and efficacy across various chemical reactions.

## 1. Introduction

In the field of contemporary chemistry, the pursuit of efficient and green catalytic systems has long been a top priority for scientific researchers. With the continuous progress of organic synthesis technology, transition metal-catalyzed reactions have become crucial in constructing complex molecular structures [1,2]. Particularly, the Tsuji–Trost reaction and Suzuki reaction, regarded as classic carbon–carbon bond-forming reactions, have extensive applications in various fields, including drug discovery, materials science, and fine chemical production [3].

The Tsuji–Trost reaction is a significant method in organic synthesis, enabling allylation and nucleophilic substitution reactions, thereby providing an effective approach for synthesizing biologically active natural products and drug molecules [4]. The Suzuki reaction, catalyzed by Pd, facilitates the cross-coupling between aryl halides and aryl boric acids to synthesize important organic molecular structures, such as biphenyls [5]. Although these two reactions hold immense value in organic synthesis, traditional catalytic systems often suffer from certain limitations. In conventional Tsuji–Trost and Suzuki reactions, the palladium catalysts typically exist in homogeneous forms and predominantly employ organic solvents [6]. Despite their high catalytic activity, these catalysts suffer from several drawbacks, including challenges in catalyst recovery, product separation, costliness, and environmental unfriendliness. To address these issues, the development of heterogeneous Pd catalysts has emerged as a promising strategy.

In recent years, the utilization of heterogeneous metal catalysts in coupling reactions has garnered significant attention from researchers due to the facile separation and recovery of end products from the reaction medium [7]. Heterogeneous metal catalysts are typically prepared by immobilizing catalytically active metal nanoparticles (MNPs) onto suitable solid supports, with various materials, such as carbon [8,9], zeolites [10,11], metal oxides [12], molecular sieves [13] ionic liquids [14], and polymers [15,16,17], being employed for this purpose. However, from the perspective of green chemistry, it is imperative to explore carrier materials that are cost-effective, non-toxic and environmentally friendly. Among many carrier materials, hydrogels have emerged as particularly promising candidates owing to their distinctive properties.

Hydrogel is a kind of polymer material with a three-dimensional network structure, which can absorb a lot of water without dissolving, thus rendering them highly suitable as catalyst carriers for aqueous-based reactions [18]. Furthermore, hydrogels possess excellent biocompatibility and stability, enabling precise control over their physical and chemical properties through composition and structural adjustments [19]. Some research findings suggest [20] that the porous nature of hydrogels can serve as a nanoreactor for organic reactions, rendering them highly suitable for Pd nanoparticle loading. The hydrogel-supported Pd catalyst overcomes the limitations of traditional systems by being water-compatible, thereby reducing reliance on harmful organic solvents. This eliminates disposal risks and potential pollution associated with traditional solvent-based catalysts used in Tsuji–Trost and Suzuki coupling reactions. Additionally, its reusability is environmentally significant as it can be reused multiple times, aligning with green chemistry principles, while reducing resource consumption and waste generation. Therefore, by fully capitalizing on the inherent advantages of hydrogels, such as their exceptional water absorption capacity, remarkable flexibility, and controllable pore structure, the immobilization of palladium catalysts onto hydrogel matrices is expected to establish an efficient, environmentally friendly, and recyclable catalytic system. This holds significant practical implications for its application in Tsuji–Trost and Suzuki reactions.

In this study, a novel P(AA-co-VI)@Pd heterogeneous catalyst was successfully prepared by using vinyl imidazole as a ligand to immobilize palladium and copolymerize with acrylic acid. The design of the P(AA-co-VI)@Pd doped gel with trace Pd aimed to optimize reactivity, stability, and performance. The selection of the P(AA-co-VI) polymer as the metal support involved incorporating a specific amount of N compound complexed with palladium, thereby enhancing catalytic activity and stability while providing flexibility and reusability. The catalyst was comprehensively analyzed and characterized using ICP-OES, SEM, EDX, Mapping, FT-IR, TGA, XRD, XPS, and TEM techniques. The results demonstrate the effective coordination bonding between Pd(II) ions and the hydrogel matrix, leading to the uniform dispersion of palladium ions on the hydrogel matrix while preventing catalyst accumulation and loss. Furthermore, the exceptional performance and reusability of the P(AA-co-VI)@Pd catalyst were verified through Tsuji–Trost and Suzuki reactions. Consequently, this study presents a novel concept along with a feasible approach for developing highly efficient, environmentally friendly, recyclable heterogeneous catalysts.

## 2. Results and Discussion

### 2.1. Characterization of P(AA-co-VI)@Pd

The composition and structure of P(AA-co-VI)@Pd were characterized by ICP-OES, SEM, EDX, Mapping, FTIR, TGA, XPS, XRD and TEM.

The Pd load was 0.012 mmol/g as measured by ICP-OES.

**Figure 1:**
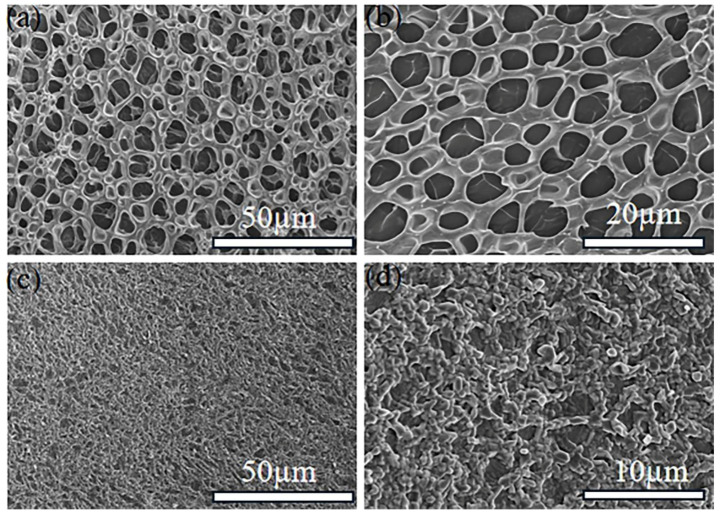
(**a**,**b**) SEM of P(AA-co-VI) hydrogels of 50 µm and 20 µm. (**c**,**d**) are SEM of the P(AA-co-VI)@Pd hydrogels.

The microscopic SEM images of P(AA-co-VI)@Pd hydrogel was characterized by studying the spatial structure and surface structure of the hydrogel. As shown in Figure 1a,b, the surface of 50 µm and 20 µm hydrogels has a uniform distribution of pore structures, and the overall crosslinking degree of the hydrogels is good, showing a porous structure. These porous structures are very conducive to water entering the hydrogel, which is conducive to the Tsuji–Trost and Suzuki reactions, and then the reaction substrate can quickly contact the catalyst, improving the catalytic performance. It can be seen from Figure 1c,d that P(AA-co-VI)@Pd hydrogel holes are covered by supported Pd, and the palladium is supported evenly, forming effective chemical crosslinking with the hydrogel, improving the supporting ability of the P(AA-co-VI)@Pd hydrogel catalyst and not easily losing its shape [21].

**Figure 2:**
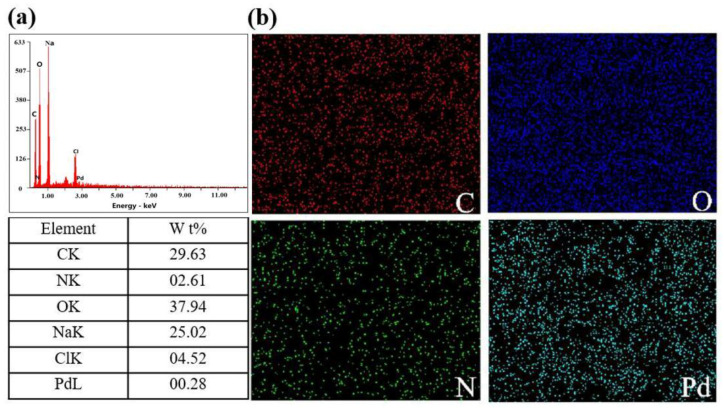
(**a**) EDS spectrum and (**b**) Mapping analysis diagram of P(AA-co-VI)@Pd.

EDS spectra and Mapping of the P(AA-co-VI)@Pd hydrogels were tested by studying the types and distribution of elements contained in the hydrogels. As shown in Figure 2a, the EDS spectrum of the hydrogel mainly contains C, N, O, and Pd elements, among which the contents of N and Pd are 2.61% and 0.28%, respectively, indicating that the palladium load is relatively uniform. As shown in Figure 2b of Mapping, it can be clearly seen that each element is very evenly distributed [22], and a small number of points in Pd element show individual agglomerations, which may be caused by insufficient stirring and ultrasound during the preparation of the P(AA-co-VI)@Pd hydrogels, so that Pd complexes are not uniformly dispersed in the whole system.

**Figure 3:**
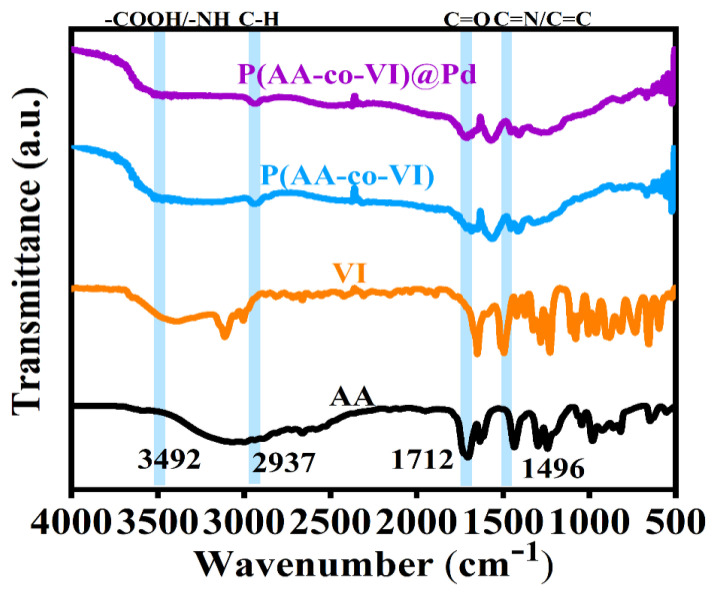
FT-IR of raw material and the P(AA-co-VI)@Pd.

FT-IR spectra of the P(AA-co-VI)@Pd hydrogels were studied by analyzing their structure and chemical bonds. As shown in Figure 3. The absorption peak at 3492 cm^−1^ corresponds to the stretching vibration of –COOH/–N–H, while the absorption peak at 2937 cm^−1^ corresponds to the C–H stretching vibration [23]. The absorption peak at 1712 cm^−1^ corresponds to the C=O stretching vibration [24]. The absorption peak at 1496 cm^−1^ corresponds to –C=N and -C=C stretching [1]. The above results show that there is no significant change in C=C and C=N on imidazole, indicating that the loaded PdCl_2_ has little fluctuation on the chemical bond, and the C=C of AA and VI are broken to form saturated C–C copolymerization, indicating that the P(AA-co-VI)@Pd hydrogel catalyst is well formed.

The thermal stability of a catalyst demonstrated by TGA across a range of temperatures has significant implications for its catalytic performance, especially in high-temperature reactions. In high-temperature reaction environments, the structure of the active sites of the catalyst is often susceptible to change. This can lead to a reduction in catalytic activity or even complete inactivation. A catalyst with strong thermal stability is able to maintain the structural integrity of its active sites at high temperatures. This is crucial because it ensures that the catalyst can continue to perform its catalytic function effectively even under extreme temperature conditions.

**Figure 4:**
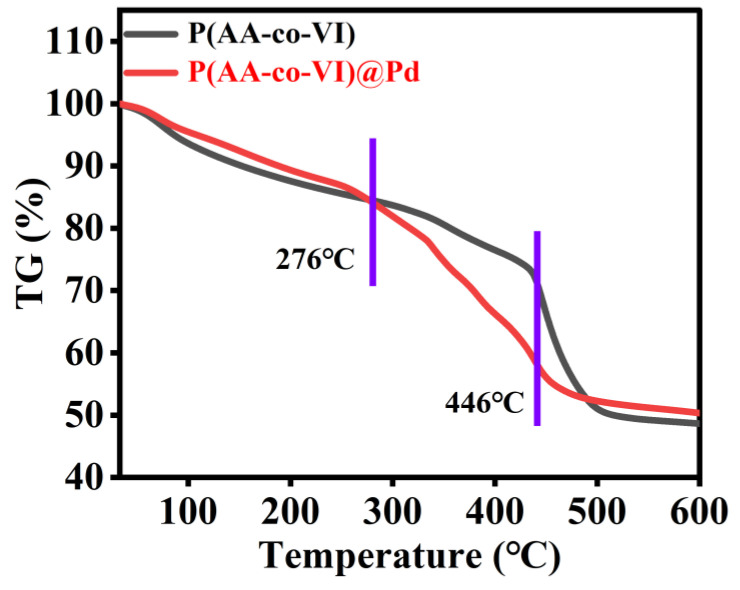
TGA of P(AA-co-VI) and P(AA-co-VI)@Pd.

Thermogravimetric analysis was performed by studying the heat resistance of the P(AA-co-VI)@Pd hydrogel. Figure 4 shows how the sample mass changes with increasing temperature. There are four stages of weight loss. The first stage is observed between 34 and 276 °C. The mass is reduced by only 15%, which is due to the presence of trace free and bound water losses in the sample. The second stage occurs between 276 and 446 °C. The mass is reduced by 11% and the degradation rate is faster. This is caused by the thermal degradation of oxygen-containing functional groups, such as carboxyl, hydroxyl and carbonyl groups, in the complex hydrogels. The third stage is between 446 and 488 °C. The mass is reduced by 19%, with the fastest degradation of the four stages [25]. This is due to the degradation of the backbone in the P(AA-co-VI)@Pd. The fourth stage is the stable stage, and the remaining part accounts for 53% of the mass percentage. Based on the above TGA data, this catalyst exhibits excellent thermal stability below 250 °C, rendering it more suitable for reactions within this temperature range.

**Figure 5:**
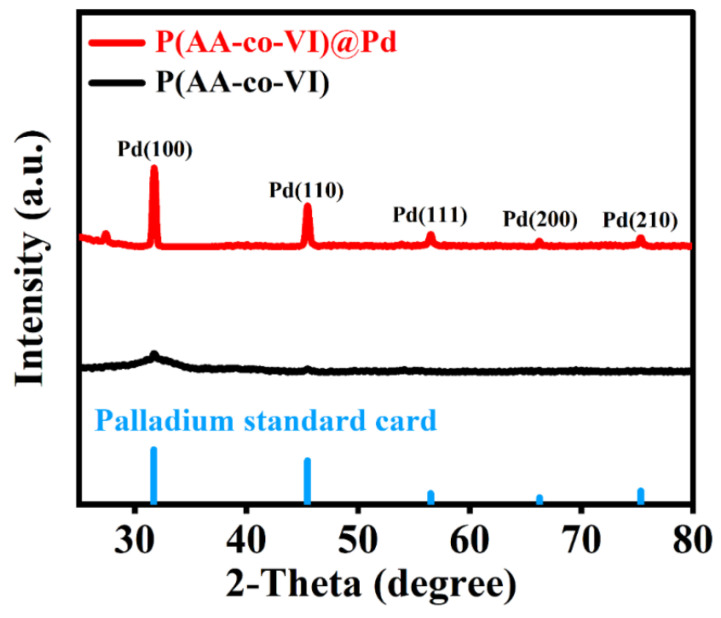
XRD of P(AA-co-VI) and P(AA-co-VI)@Pd.

The crystal structure and crystallinity of Pd in the P(AA-co-VI)@Pd hydrogel was studied by XRD. As can be seen from Figure 5, corresponding peaks appear in the XRD patterns of all samples at 31.7, 45.5, 56.5, 66.2, and 75.3, corresponding to the (100), (110), (111), (200), and (210) planes of Pd [26], respectively, and these peaks correspond to the characteristic diffraction peaks. The position of the peak corresponds to the Pd standard card at the bottom one-by-one, which further indicates that the Pd complex is successfully combined with acrylic acid. All the diffraction peaks are consistent with the diffraction peaks of Pd, and no other crystal diffraction peaks appear. It can also be seen from Figure 5 that Pd peak intensity is high, indicating that it has a high degree of crystallinity. In the XRD of P(AA-co-VI)@Pd, the peak value is reduced, which is caused by the decrease of Pd proportions. There is no Pd peak in the P(AA-co-VI) hydrogel.

**Figure 6:**
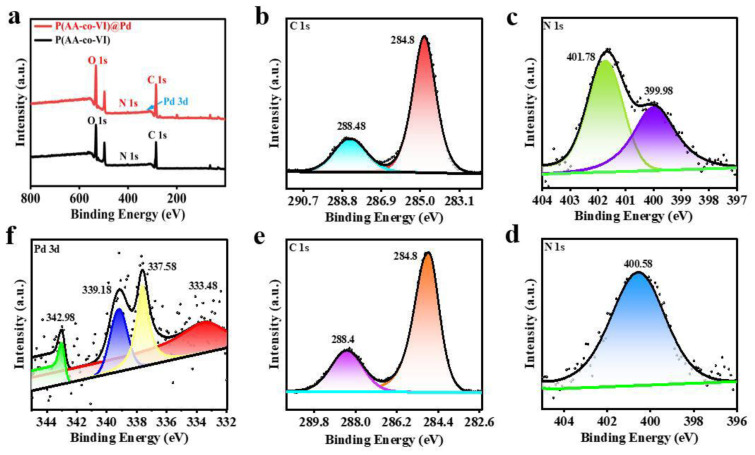
(**a**) XPS scan image and the high-resolution spectroscopy of C 1s (**b**), N 1s (**c**), N 1s (**d**), C 1s (**e**), and Pd3d (**f**) of the catalyst P(AA-co-VI) and P(AA-co-VI)@Pd.

The surface composition of the catalyst was characterized by XPS. The full-scan XPS in Figure 6a shows P(AA-co-VI) and P(AA-co-VI)@Pd, with corresponding signals for C, N, and C, N, and Pd elements, respectively. The spectrum of C 1s in Figure 6b can deconvolve two peaks, 288.48 eV and 284.8 eV, respectively. The N 1s spectrum in Figure 6c has two peaks at 401.78 eV and 399.98 eV. Figure 6d is the P(AA-co-VI)@Pd N 1s spectrum with only one peak at 400.58 eV. The P(AA-co-VI)@Pd load is successful because peak N is shifted. Figure 6e C 1s spectrum has two peaks of 288.4eV and 284.8 eV. It is basically the same as Figure 6b. Figure 6f shows that the peak values associated with Pd(0) in the catalyst are 333.48 eV and 339.18 eV, and the peak values associated with Pd(II) are 337.58 eV and 342.98 eV, corresponding to the Pd3d5/2 and Pd3d2/3 orbits [27], respectively. This indicates that Pd(II) is successfully fixed and partially reduced to Pd(0) in the composite material. The two peaks with binding energy of 342.98 eV and 337.58 eV in the figure are consistent with Pd(0); the two peaks of 339.18 eV and 333.48 eV are confirmed as Pd(II); and the peak area of Pd(0) is larger than that of Pd(II) [28]. This indicates that Pd(II) in the recovered catalyst may be in situ reduced to Pd(0). During this process, the surface properties significantly influence catalytic activity. Regarding the Pd(II) to Pd(0) transitions: A larger surface area provides more active sites for reactant adsorption and stabilization of the reaction intermediates during the conversion of Pd(II) to Pd(0). The presence of different elements or compounds alters the electronic and chemical properties, modifying the interaction with reactants. Additionally, a shift in surface potential accompanying the redox change can regulate reactant adsorption and dissociation, making the catalyst more favorable for specific reactions by adjusting the interaction energy.

**Figure 7:**
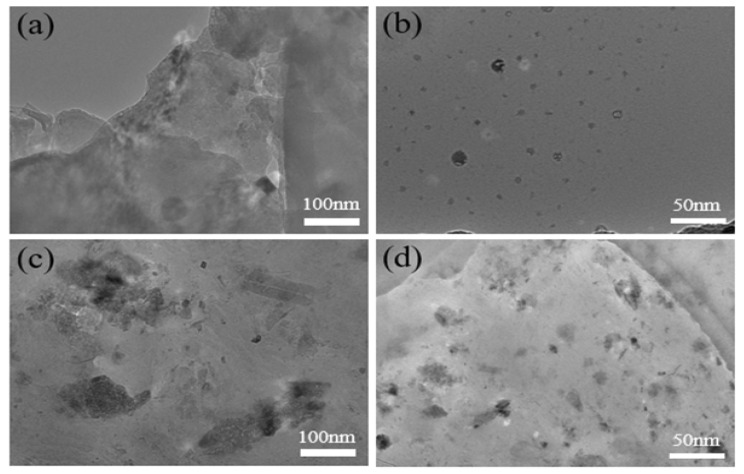
The TEM micrographs of P(AA-co-VI)@Pd: (**a**,**b**) after 1st time recovered, (**c**,**d**) after 6th time recovered.

Under TEM, as shown in Figure 7a,b, the transmission diagram of the P(AA-co-VI)@Pd hydrogel catalyst after the first cycle shows that the dispersion is still relatively uniform, and a small amount of Pd is leachable. Figure 7c,d are the sixth cycle, and the leaching of Pd gradually increases, indicating that the more cycles, the more leaching. However, the agglomeration of Pd was only a small amount, and the distribution was still uniform.

### 2.2. Swelling Properties

**Figure 8:**
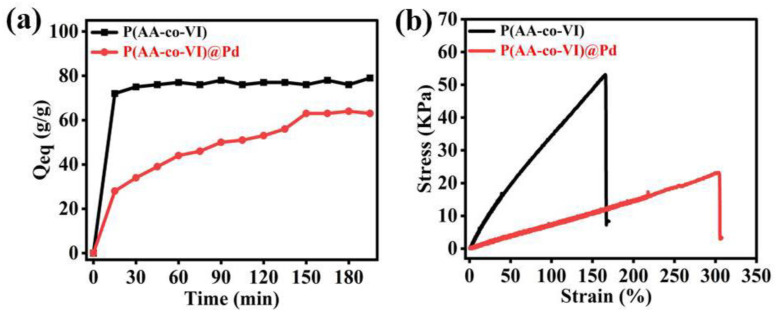
Swelling and mechanical properties of (**a**,**b**) P(AA-co-VI) and P(AA-co-VI)@Pd.

The P(AA-co-VI)@Pd is easy to absorb water and swell in water, so test the swelling performance of the P(AA-co-VI)@Pd hydrogel. As can be seen from Figure 8a, the water absorption of the P(AA-co-VI)@Pd hydrogel reached 28 g/g at 5 min, and 76 g/g at 150 min, which was in adsorption equilibrium. This indicates that the water absorption rate of the hydrogel is very fast, and it can reach saturation in a very short time. This is because the P(AA-co-VI)@Pd hydrogel has a three-dimensional network structure, which is conducive to the entry of water molecules into the interior, and there are a large number of -COOH, -OH, and other groups in the hydrogel, which are easy to form hydrogen bonds with water molecules and can absorb a large amount of water in a short time.

### 2.3. Mechanical Properties

The mechanical properties of the P(AA-co-VI)@Pd hydrogels are tested with the aim of ensuring that the material has the ideal mechanical strength when using aqueous solvents. Figure 8b shows the strain–strain diagram of the P(AA-co-VI)@Pd hydrogel tested on a tension machine. Carefully observing this figure, it can be found that the strength of the P(AA-co-VI)@Pd hydrogel will continue to increase with the gradual increase of strain. Its maximum value is 23 KPa when the strain reaches 304%.

### 2.4. Application of P(AA-co-VI)@Pd in Tsuji–Trost

Firstly, the catalytic activity of the P(AA-co-VI)@Pd catalyst in a Tsuji–Trost reaction was studied. Using cinnamyl acetate (0.088 g, 0.5 mmol) and NaBPh_4_ (0.342 g, 1.0 mmol) as reaction substrates, the reaction conditions were optimized by studying the effects of different solvents, the amount of catalyst load and reaction time, as shown in Table 1. First, we screened different solvents. In ethanol, the hydrogel did not swell, so the reaction could not be carried out. Substituting with other solvents results in a suboptimal yield. When water was used as a solvent, the reaction reached equilibrium at 2 h without nitrogen protection, and the yield was 94%. In addition, less solvent leads to a larger contact area for a complete reaction, so we choose to use only 5 mL. Subsequently, we found that the P(AA-co-VI)@Pd catalyst showed better activity in the Tsuji–Trost reaction than other reported Pd catalysts. The effect of different amounts of catalyst on the reaction was 94% yield at 10 mg, 93%, 90%, and 95% conversion at 5 mg, 15 mg and 20 mg, respectively. The results showed that, at 5 mg, the reaction time increased to 3 h, and the yield was not very high. The yield of the P(AA-co-VI)@Pd catalyst at 15 mg and 20 mg is relatively large, and the yield is not different. A lower catalyst load will result in complete conversion, and increasing the catalytic dose does not significantly improve conversion efficiency. At the same time, the increasing reaction time was investigated, and there was no significant change in the reaction.

Therefore, the optimal conditions for Tsuji–Trost reaction are as follows: Cinnamyl acetate (0.088 g, 0.5 mmol), NaBPh_4_ (0.342 g, 1.0 mmol), and 10 mg (0.12 µmol Pd) of P(AA-co-VI)@Pd catalysts in 5 mL H_2_O were reflowed at 80 °C for 2 h without nitrogen protection.

### 2.5. Application of the P(AA-co-VI)@Pd in Suzuki

Strongly encouraged by the results of Tsuji–Trost reaction, the application of the P(AA-co-VI)@Pd catalyst in a Suzuki reaction was investigated by this paper. In this process, only 10 mg of P(AA-co-VI)@Pd (0.12 µmol Pd) was used as a catalyst to focus on the reaction between iodobenzene and phenylboronic acid.

The use of water as a solvent in the relevant reactions has been explicitly mentioned previously in the literature. In this study, a detailed comparative analysis of the amounts of aqueous solvents and catalysts has been carried out. After a series of rigorous experimental verification, the results show that the hydrogel properties of the P(AA-co-VI)@Pd catalyst, and therefore the expected reaction in water, does occur. However, what was particularly surprising was that when the reaction was reflowed in 80 °C waters, it was successfully completed in just 1 h. At this point, the yield of the product was as high as 99%, a remarkable result, which is shown in Table 2.

Further, the reactions of different substituted aryl iodides and bromides with phenylboronic acid were studied; the results showed that the situation was similar to the previous situation. The specific data are presented in Table 3. Regardless of the properties of the substituents on the benzene ring, the yield of the biphenyl products is always maintained at a high level. Under all experimental conditions, P(AA-co-VI)@Pd showed excellent catalytic activity. This finding provides strong support for the further development and application of a Suzuki reaction. In the future, researchers can further explore the catalyst performance in more complex reaction systems, as well as its adaptability to different types of substrates. At the same time, further optimization and improvement of the catalyst can also be considered to improve its catalytic efficiency and selectivity and bring more innovations and breakthroughs in the field of organic synthesis. It is believed that, with the deepening of research, the application prospect of the P(AA-co-VI)@Pd hydrogel catalyst in a Suzuki reaction and other related reactions will be broader.

### 2.6. Recovery of the P(AA-co-VI)@Pd Catalyst

**Figure 9:**
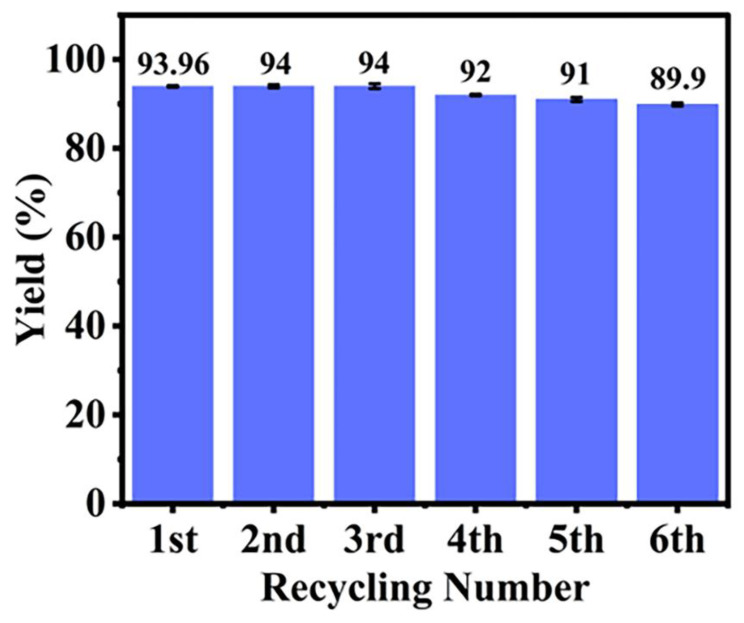
Recovery of P(AA-co-VI)@Pd in the Tsuji–Trost reaction. Reaction conditions: NaBPh_4_ (0.342 g, 1.0 mmol), P(AA-co-VI)@Pd catalyst (10 mg, 0.012 µmol Pd), cinnamyl acetate (0.088 g, 0.5 mmol), and water (5 mL) reflux at 80 °C.

As shown in Figure 9, after the first Tsuji–Trost reaction, the catalyst was filtered with a small number of multiple times of water before being put into a new reaction. The same regeneration process was repeated six times with no significant loss of activity. As a result, the separated and washed catalyst can be reused. This washed step effectively removes waste inorganic material from the catalyst, which initially hinders the active site, favoring subsequent reactions and cycling. As shown in Figure 9, the yield can still reach 89.9% after six cycles, indicating that the P(AA-co-VI)@Pd catalyst is relatively stable, and the supported palladium is not easy to fall off. However, the use of tridentate ligands can be considered in future studies. Considering environmental friendliness and non-toxicity, natural polymers can be selected as catalyst carriers in future studies. This study shows that heterogeneous catalysts are superior to homogeneous catalysts in terms of recyclability and reusability. It is hoped that more natural polymers can be used as carriers of heterogeneous catalysis in future studies.

### 2.7. Reaction Mechanism of Tsuji–Trost and Suzuki

**Figure 10:**
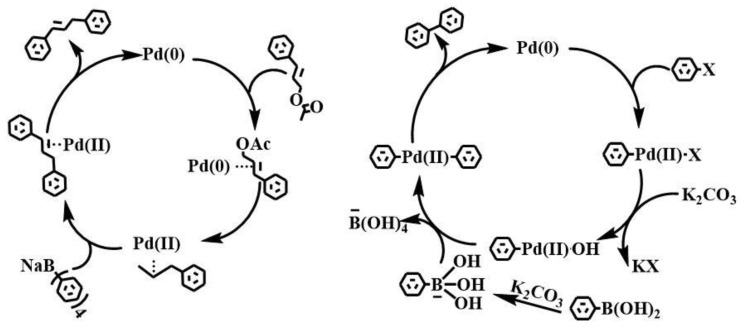
Mechanism of the Tsuji–Trost and Suzuki reactions.

The cross-coupling reaction strictly follows a specific catalytic cycle, which is clear and orderly. As shown in Figure 10, the first step of oxidation to add organic halides occurs in the Pd(0) complex. During this process, the organic halide interacts with the Pd(0) complex, kicking off the whole reaction. Then, with the help of the base, the critical step of metal conversion takes place [29]. This step is crucial because it directly affects the process of the reaction and the formation of the final product. In different cross-coupling reactions, the metal conversion steps may vary considerably depending on the reagent used and the reaction conditions. For example, when different bases are used, their promotion of metal conversion may be different, affecting the rate and selectivity of the reaction. Similarly, changes in conditions, such as reaction temperature and pressure, can have a significant impact on metal conversion. Finally, the formation of new C-C bonds is eliminated by reduction while regenerating the catalyst. This step marks the end of a complete catalytic cycle, while also setting the stage for the next round of reaction.

In these steps, the first step of oxidation, to add organic halides, and the last step of reduction, to eliminate the formation of new C−C bonds, are common features [30]. These two steps have been well understood through extensive experimental research. Experimental studies can directly observe the phenomena of reactions and the formation of products and provide an important basis for theoretical analysis. In this paper, the catalyst is carried out in water, so that its hydrogel catalyst swelling increases its specific surface area volume and more active sites. The reaction is accelerated, and the catalytic efficiency is improved. However, it is precisely because of the diversity of metal conversion steps that cross-coupling reactions show different characteristics and application values in different systems. Future studies can further explore the mechanism and influencing factors of the metal conversion steps to optimize the conditions of the cross-coupling reaction, improve the efficiency and selectivity of the reaction, and bring more innovations and breakthroughs to the field of organic synthesis.

### 2.8. Comparison of Catalytic Activity of P(AA-co-VI)@Pd Catalyst with Other Reported Catalysts in Tsuji–Trost and Suzuki Reactions

The performance of four different palladium catalysts in previous studies was compared with that of the P(AA-co-VI)@Pd catalyst prepared in this study in Tsuji–Trost and Suzuki reactions, as shown in Table 4. It can be observed from the data that the P(AA-co-VI)@Pd catalyst achieved a remarkable yield of up to 94% within a reaction time of only 2 h, surpassing other catalysts significantly in the Tsuji–Trost reaction. By contrast, the other four catalysts yielded 99%, 98%, 80%, and 85% with reaction times of 12, 12, 48, and 4 h, respectively. Moreover, the Suzuki reaction has excellent catalytic performance, reaching a yield of 99% within one hour, compared with the reaction time of the other four catalysts of 6, 2, 1, and 4 h, respectively, and the yield of 99%, 96%, 73%, and 98%. In addition, TON and TOF are relatively larger than other references, and a larger TON value indicates that each active center can convert more reactants; that is, reactants can participate in the reaction more fully, and a larger TOF means that more reactant molecules can be converted per active center per unit time, resulting in a significantly higher rate of product formation. Overall, our study demonstrates that the P(AA-co-VI)@Pd catalyst exhibits superior catalytic efficiency within a relatively short reaction time when compared to the existing literature.

## 3. Conclusions

In this study, the P(AA-co-VI)@Pd catalyst was prepared by heat-initiated polymerization, starting with the formation of a complex between vinyl imidazole and palladium chloride, followed by the addition of neutralized acrylic acid (AA), crosslinking agent, and initiator. The catalyst’s structure and morphology were characterized by using various techniques to confirm the immobilization of palladium onto hydrogel matrices for aqueous-based reactions.

Under optimized conditions using water as the solvent, the P(AA-co-VI)@Pd (10 mg) catalyst exhibited remarkable catalytic activity, achieving a Tsuji–Trost reaction yield of 94%. Moreover, it demonstrated excellent reusability and environmental friendliness by maintaining good activity and structural integrity for six consecutive cycles with a yield of 89.9%, making it suitable for sustainable synthesis. Additionally, it displayed outstanding catalytic performance in Suzuki reactions with a conversion rate of 100% and a yield of 99% within just one hour.

In conclusion, the Pd catalyst embedded in the P(AA-co-VI)@Pd hydrogel exhibits remarkable catalytic activity, facile filtration and separation, as well as excellent reusability, while complying with environmental regulations. Therefore, it presents a promising alternative for relevant reactions. However, this study has limitations in investigating catalyst efficiency across different reaction media. In our forthcoming research, we aim to comprehensively examine the catalyst’s performance in diverse solvents, pH conditions, and temperature ranges. For instance, we will evaluate its activity in both polar and non-polar solvents under acidic, basic, and neutral pH environments. By undertaking this future research endeavor, we can address existing gaps and provide more comprehensive insights for the development and application of our catalyst system.

## 4. Materials and Methods

### 4.1. Materials

Petroleum ether (PE), N, N-methylene bis-acrylamide (MBA), and ammonium persulfate (APS) were purchased from Tianjin Chemical Reagent Company, Tianjin, China. 1-vinylimidazole (VI), iodobenzene, phenylboronic acid, p-iodotoluene, potassium carbonate (K_2_CO_3_), bromobenzene, p-bromotoluene, and 4-iodonitrobenzene were purchased from Shanghai McLean Biochemical Technology Co., Ltd., Shanghai, China. Ethyl acetate (EA) was purchased from Beijing Yinokai Technology Co., Ltd., Bejing, China. Sodium tetraphenylborate, acrylic acid (AA), and sodium hydroxide (NaOH) were purchased from Shandong Keyuan Biochemical Co., Ltd., Jinan, China. Cinnamyl acetate was purchased from Shanghai Kelin Technology Co., Ltd., Shanghai, China; anhydrous ethanol (C_2_H_5_OH) was purchased from Shanghai Kelin Technology Co., Ltd., Shanghai, China. Palladium chloride (PdCl_2_) was purchased from Changzhou Boca Chemical, Changzhou, China. Silica gel powder was purchased from Qingdao Oceanview Chemical Co., Ltd., Qingdao, Chengdu, China. Hydrochloric acid (HCl) was purchased from Sichuan Xilong Science Co., Ltd., Chengdu, China.

### 4.2. Preparation of (P(AA-co-VI) @Pd)

**Figure 11:**
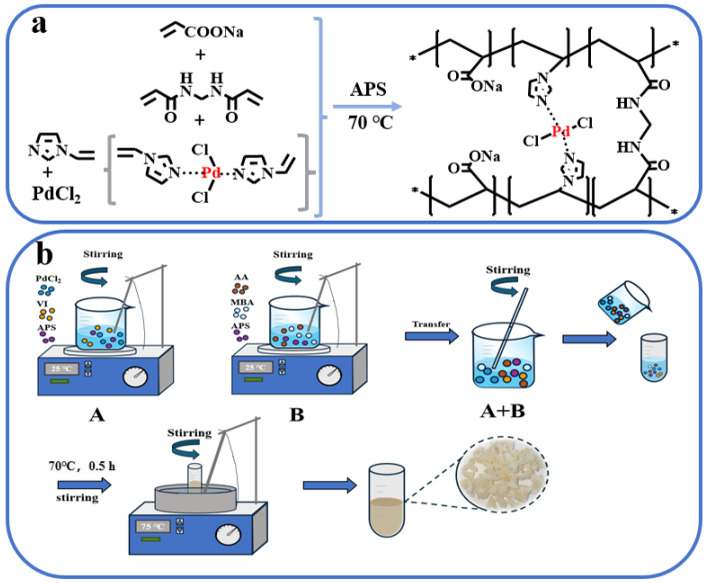
Chemical structure formula (**a**) and preparation process (**b**) of the P(AA-co-VI)@Pd catalyst.

The chemical structure formula and preparation process chart is shown in Figure 11. First, accurately weigh 2.5 mL AA solution, add 2.5 mL NaOH solution with a concentration of 5 M to it, mix it thoroughly and let it cool to room temperature. Then, 0.99 wt% (both mass percentages of AA) MBA and 0.25 wt% APS was added, and then 5 mL distilled water was added for ultrasonic treatment for 10 min to ensure full dissolution, which was recorded as solution A.

Additionally, 5 mg of palladium chloride was added to 10 mL of 0.5 mol/L hydrochloric acid solution until completely dissolved. Then 90 μL vinyl imidazole was added and the complex reaction was carried out for 5 min by magnetic stirring. After that, 0.25 wt % APS was added and magnetically stirred again for 5 min, which was recorded as solution. Finally, solution A and solution B were mixed, and ultrasonic treatment was performed for 30 min. The mixed solution was transferred into a glass tube and placed in a constant temperature water bath stirrer, stirring and heating at 70 °C until a gel was formed. Take out the prepared hydrogel and put it in a blast drying oven for drying [39].

### 4.3. Characterization

The test was carried out by KBr compression method through the equipment of BRUKER company in Germany. The spectrum ranges from 4000 to 500 cm^−1^; such a wide range can capture the vibration information of various chemical bonds in the catalyst, providing important clues for determining its chemical structure. The resolution is 2 cm^−1^, which can fine distinguish different absorption peaks, and the 8 scanning times ensure the accuracy and stability of the test results.

The change of sample mass with temperature or time was observed by HITACHI STA7300 TGA under N_2_; condition at a gas flow rate of 200 mL/min and a heating rate of 10 °C/min. The thermal stability of the P(AA-co-VI)@Pd catalyst can be determined by a TGA test.

The ICP-OES method is used to accurately determine the content of palladium in the catalyst by Agilent’s instrument. This method has high sensitivity and accuracy.

The surface morphology of the sample was studied by scanning electron microscope (SU8010) (SEM). The sample was freeze-dried and sprayed with a gold coating and tested at an acceleration voltage of 20 KV.

The dispersion spectroscopy (EDX) and Mapping techniques by HITACHI Instruments in Japan were used to test the dispersion of palladium. EDX can provide information about the elemental composition, and Mapping can visually show the distribution of Pd in the catalyst.

XPS testing on Thermo Fisher Scientific K-Alpha instrument can provide insight into the chemical bond changes on the catalyst surface. XPS can provide information about the chemical state and valence state of the element, help to reveal the mechanism of the catalyst in the reaction process, and provide a theoretical basis for further improvement of the catalyst.

Cu K was used as the X-Ray source by X-Ray diffractometer (D8 Advance), the X-Ray wavelength was 0.154nm, the crystal phase of the sample was observed, and the powder X-Ray diffraction (XRD) pattern of the sample was collected.

Transmission electron microscopy (TEM) through the Japanese Electronic instrument was used to observe the leaching of palladium. TEM has a high resolution and can clearly show the microstructure of the catalyst, which provides a basis for improving the service life of the catalyst.

### 4.4. Measurement of Swelling Ratio

Dry P(AA-co-VI)@Pd hydrogel is ground and weighed to 0.1g in sufficient distilled water, which is a common method for determining water absorption of hydrogel. The hydrogel continues to absorb water over time. Weigh the hydrogel after straining it every 10 min and carefully removing excess water, a process that requires careful handling to ensure the accuracy of the measurement [40]. By calculating water absorption, you can obtain an idea of how absorbent the hydrogel is. The calculation formula reflects the hydrogel’s ability to absorb water as a percentage of its initial dry state. Favorable reaction in aqueous solvent. The formula is as follows [41].

(1) $$Water\;absorption\left(\%\right)=\frac{W_{t}-W_{0}}{W_{0}}\times 100$$

### 4.5. Measurement of Mechanical Properties

The mechanical test was carried out by electric universal tensile testing machine at room temperature, and the length of the instrument was fixed at 2 cm. A rectangular sample with a thickness of 2.0 mm (30 mm long, 10 mm wide) was tested at a stretch rate of 50 mm/min. Each sample set consists of 3–5 hydrogel samples for testing. Before the test, the hydrogel is removed from a glass plate or centrifuge tube and the surface is coated with dimethyl silicone oil to inhibit the evaporation of water. The strength, elastic modulus, and other mechanical parameters of hydrogels can be evaluated by recording stress, strain, and other data during stretching.

### 4.6. P(AA-co-VI)@Pd General Steps for Tsuji–Trost

NaBPh_4_ (0.342 g, 1.0 mmol), P(AA-co-VI)@Pd (0.12 µmol Pd), cinnamyl acetate (0.088 g, 0.5 mmol), and water (H_2_O) (5 mL) were successively added to the round-bottomed flask. At this time, the whole reaction system was in the state of no nitrogen protection. The mixture was placed at 80 °C (reflux) and continued to react by magnetic agitation for 2 h, during which the reaction was monitored in real time by thin layer chromatography (TLC). When the reaction is complete, the mixture is cooled to room temperature. Next, the catalyst and the reaction mixture are separated by filtration and the P(AA-co-VI)@Pd catalyst is thoroughly rinsed with H_2_O and ethyl acetate (EA). The catalyst is then put into a new reaction to continue the reaction. The reaction mixture is then extracted with a saturated salt solution (100 mL) and EA to obtain a suspension. Then anhydrous magnesium sulfate was used to dry the suspension. After the solvent is removed by a decompression operation, the crude product needs to be further purified. The crude product is purified by silica gel column chromatography (petroleum ether:ethyl acetate = 150:1) to obtain a colorless oil-like product. The ^1^H NMR and ^13^C NMR nuclear resonance numbers of the product are shown on the attached page.

(E)-prop-1-ene-1,3-diyldibenzene:^1^H NMR (600 MHz, Chloroform-d): δ 7.35–7.13 (m, 10H), 6.42 (dt, *J* = 15.7, 1.5 Hz, 1H), 6.32 (dt, *J* = 15.8, 6.8 Hz, 1H), 3.51 (dd, *J* = 6.8, 1.4 Hz, 2H).^13^C NMR (151 MHz, Chloroform-d) δ 140.44, 137.77, 131.39, 129.49, 128.98, 128.84, 127.40, 126.49, 126.44, 39.64 (Figures S1 and S2).

### 4.7. P(AA-co-VI)@Pd General Steps for Suzuki

Add phenylboronic acid (0.74 mmol), 10 mg P(AA-co-VI)@Pd (0.12 µmol Pd), potassium carbonate (0.94 mmol), iodobenzene (0.5 mmol), and H_2_O solvent, in turn, to a 5 mL round-bottomed bottle. In the absence of nitrogen protection, this mixture was placed at 80 °C (in a reflux state) and continued for 1 h by magnetic agitation. During this process, the reaction is monitored in real time using thin layer chromatography (TLC).

When the reaction is complete, the mixture is cooled to room temperature. Next, the P(AA-co-VI)@Pd catalyst and the reaction mixture are filtered and separated, and the P(AA-co-VI)@Pd catalyst is thoroughly cleaned with H_2_O. Then, the catalyst was put into a new reaction to continue the reaction. The reaction mixture is then extracted with a saturated brine solution (100 mL) and ethyl acetate (EA) to obtain a suspension, which is then dried with anhydrous magnesium sulfate. Finally, the solvent was removed by a decompression operation, and the crude product was further purified with petroleum ether:ethyl acetate = 100:1 as eluent. ^1^H NMR and ^13^C NMR nuclear resonance data for this product are shown on the attached page.

Biphenyl: ^1^H NMR (600 MHz, Chloroform-d): δ 7.62–7.58 (m, 2H), 7.44 (d, *J* = 15.4 Hz, 1H), 7.35 (t, *J* = 7.4 Hz, 1H). ^13^C NMR (151 MHz, Chloroform-d) δ 141.39, 128.90, 127.39, 127.32 (Figures S3 and S4)

4-methyl biphenyl: ^1^H NMR (600 MHz, Chloroform-d): δ 7.59 (d, J = 8.1 Hz, 2H), 7.51 (d, *J* = 8.1 Hz, 2H), 7.44 (d, *J* = 7.7 Hz, 2H), 7.33 (t, *J* = 7.4 Hz, 1H), 2.41 (s, 3H). ^13^C NMR (151 MHz, Chloroform-d) δ 141.55, 138.75, 137.40, 129.86, 129.09, 127.55, 127.38, 21.48 (Figures S5 and S6).

4-Nitrobiphenyl: ^1^H NMR (600 MHz, Chloroform-d): δ 8.25 (s, 0H), 7.75 (s, 2H), 7.62 (s, 2H), 7.50 (s, 3H).^13^C NMR (151 MHz, Chloroform-d) δ 147.03, 129.09, 128.85, 127.74, 127.33, 124.05 (Figures S7 and S8).

## Figures and Tables

**Table 1:** The optimization of Tsuji–Trost reaction conditions.

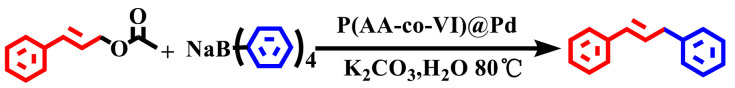					
**Entry ^a^**	**Catalyst (mg)**	**Solvent (mL)**	**Temp. (** **°C)**	**Time (h) ^b^**	**Yield (%) ^c^**
1	10	H_2_O (5)	80	2	94
2	10	CH_3_CH_2_OH (5)	80	12	trace
3	10	H_2_O (5)	40	2	45
4	5	H_2_O (5)	80	3	93
5	15	H_2_O (5)	80	2	90
6	20	H_2_O (5)	80	2	95

^a^ Reaction condition: cinnamyl acetate (0.088 g,0.5 mmol), NaBPh_4_(0.342 g,1.0 mmol), P(AA-co-VI)@Pd catalyst (10 mg), H_2_O (5 mL), and 2 h; ^b^ Detected by TLC; ^c^ Isolated yield.

**Table 2:** The optimization of Suzuki reaction condition.

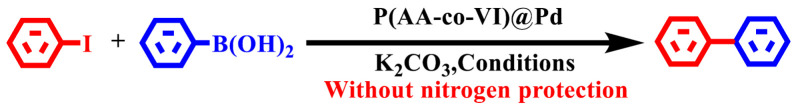					
**Entry ^a^**	**Catalyst (mg)**	**Solvent (mL)**	**Temp. (** **°C)**	**Time (h) ^b^**	**Yield (%) ^c^**
1	15	H_2_O (5)	80	1	99
2	15	CH_3_CH_2_OH (5)	80	12	Trace
3	15	CH_3_CH_2_OH/H_2_O (1:1)	80	4	99
4	15	H_2_O (5)	40	12	Trace
5	5	H_2_O (5)	80	3	99
6	10	H_2_O (5)	80	1	99
7	20	H_2_O (5)	80	1	99

^a^ Reaction condition: iodobenzene (0.102 g, 0.5 mmol), Ph B(OH)_2_ (0.0902 g, 0.74 mmol), K_2_CO_3_ (0.1297 g, 0.94 mmol), P(AA-co-VI)@Pd catalyst (10 mg), H_2_O (5 mL), and 1 h; ^b^ Detected by TLC; ^c^ Isolated yield.

**Table 3:** Different reaction substrates.

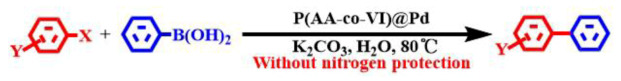			
**Entry ^a^**	**Substrate**	**Time (h) ^b^**	**Yield (%) ^c^**
1	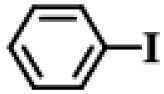	1	99
2	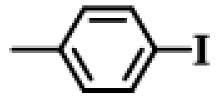	0.5	96
3	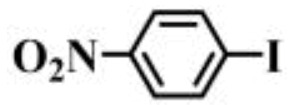	1	95
4	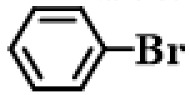	1	98
5	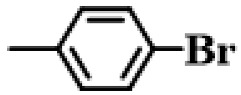	1	97

^a^ Reaction condition: Aryl halides (0.5 mmol), Ph B(OH)_2_ (0.74 mmol), K_2_CO_3_ (0.94 mmol), P(AA-co-VI)@Pd catalyst (10 mg), and H_2_O (5 mL); ^b^ Detected by TLC; ^c^ Isolated yield.

**Table 4:** Comparison of catalytic efficiency between this paper and other catalysts.

Entry	Catalyst	Solvent (mL)	Temp. (°C)	Time (h)	Yield (%)	TON	TOF (h^−1^)	Reference
1	P(AA-co-VI)@Pd	H_2_O	80	2	94	7.83 × 10^6^	3.92 × 10^6^	This work
2	Pd(PPh_3_)_4_	H_2_O	100	12	92	23	1.91	[31]
3	Pd(PPh_3_)_4_	DCM	75	12	96	24	2	[32]
4	Pd(PPh_3_)_2_Cl_2_	dioxane	reflux	18	92	18.4	1.53	[33]
5	Pd(Ph CN)_2_Cl_2_	DCM	r.t	4	85	17	4.25	[34]
6	P(AA-co-VI)@Pd	H_2_O	80	1	99	8.25 × 10^6^	8.25 × 10^6^	This work
7	Pd@IO-chitosan	H_2_O	100	6	99	1.8 × 10^4^	3 × 10^3^	[35]
8	OCMCS-SB-Pd(II)	EtOH:H_2_O = 3:2	50	2	96	208.8	104.4	[36]
9	Fe_3_O_4_/PEG/Pd	EtOH:H_2_O = 1:1	60	1	73	1.8 × 10^4^	1.8 × 10^4^	[37]
10	PdCl_2_(Ln@β-CD)	TBAB	90	4	98	9.8 × 10^3^	2.45 × 10^3^	[38]

## Data Availability

The original contributions presented in the study are included in the article/Supplementary Materials, further inquiries can be directed to the corresponding authors.

## Supplementary Materials

The following supporting information can be downloaded at: https://www.mdpi.com/article/10.3390/gels10120758/s1, Figure S1: ^1^H NMR (600 MHz, Chloroform-*d*) δ 7.35 – 7.13 (m, 10H), 6.42 (dt, *J* = 15.7, 1.5 Hz, 1H), 6.32 (dt, *J* = 15.8, 6.8 Hz, 1H), 3.51 (dd, *J* = 6.8, 1.4 Hz, 2H); Figure S2: ^13^C NMR (151 MHz, Chloroform-d) δ 140.44, 137.77, 131.39, 129.49, 128.98, 128.84, 127.40, 126.49, 126.44, 39.64; Figure S3: ^1^H NMR (600 MHz, Chloroform-d) δ 7.62–7.58 (m, 2H), 7.44 (d, J = 15.4 Hz, 1H), 7.35 (t, J = 7.4 Hz, 1H); Figure S4: ^13^C NMR (151 MHz, Chloroform-d) δ 141.39, 128.90, 127.39, 127.32 (3) 4-methylbiphenyl: 1H NMR and 13C NMR spectrum (in CDCl3); Figure S5: ^1^H NMR (600 MHz, Chloroform-d) δ 7.59 (d, J = 8.1 Hz, 2H), 7.51 (d, J = 8.1 Hz, 2H), 7.44 (d, J = 7.7 Hz, 2H), 7.33 (t, J = 7.4 Hz, 1H), 2.41 (s, 3H); Figure S6: ^13^C NMR (151 MHz, Chloroform-d) δ 141.55, 138.75, 137.40, 129.86, 129.09, 127.55, 127.38, 21.48; Figure S7: ^1^H NMR (600 MHz, Chloroform-d) δ 8.25 (s, 0H), 7.75 (s, 2H), 7.62 (s, 2H), 7.50 (s, 3H); Figure S8: ^13^C NMR (151 MHz, Chloroform-d) δ 147.03, 129.09, 128.85, 127.74, 127.33, 124.05.
