# Supplementary material for: Preparation and Characterization of Poly(acrylic acid-co-vinyl imidazole) Hydrogel-Supported Palladium Catalyst for Tsuji–Trost and Suzuki Reactions in Aqueous Media
Source: Gels. 2024 Nov 23;10(12):758. doi: 10.3390/gels10120758 (PMC11675493; doi:10.3390/gels10120758)
Supplement: Supplementary file 1 [file gels-10-00758-s001.zip › gels-3303369-supplementary.pdf]

Supporting Information

^{1}H NMR, ^{13}C NMR and spectroscopic data of the Tsuji-Trost and Suzuki reaction products

Characterization of the product obtained from the Tsuji-Trost and Suzuki reaction was performed using ^{1}H NMR and ^{13}C NMR spectroscopy.

(1) (E)-prop-1-ene-1,3-diyldibenzen :^{1}H NMR and ^{13}C NMR spectrum (in $CDCl_3$):

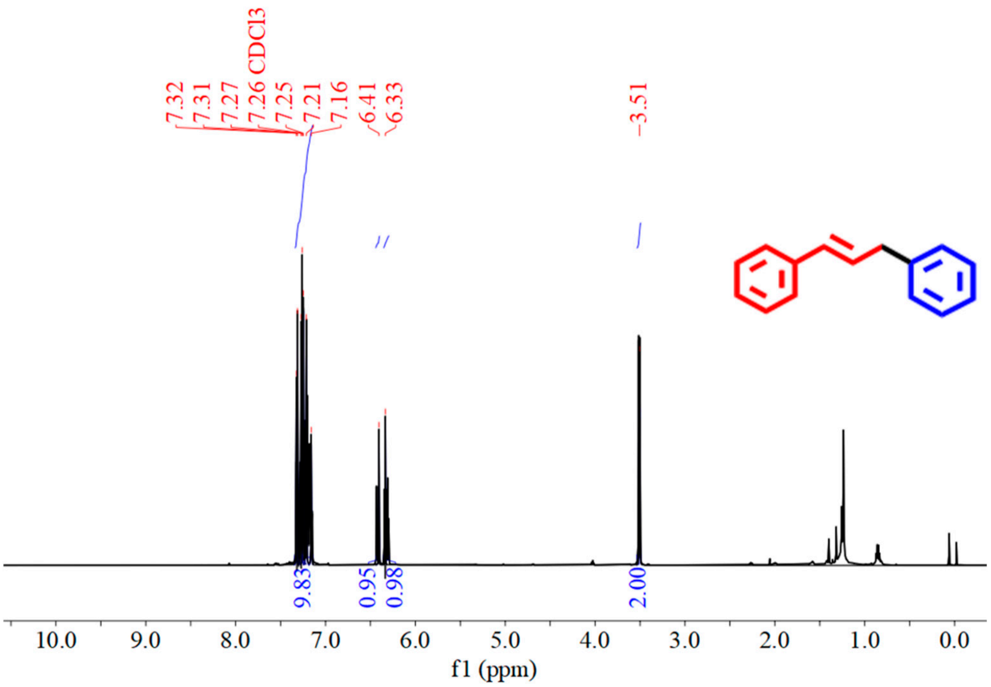

Figure S1 ^{1}H NMR (600 MHz, Chloroform-*d*) δ 7.35 – 7.13 (m, 10H), 6.42 (dt, J = 15.7, 1.5 Hz, 1H), 6.32 (dt, J = 15.8, 6.8 Hz, 1H), 3.51 (dd, J = 6.8, 1.4 Hz, 2H)

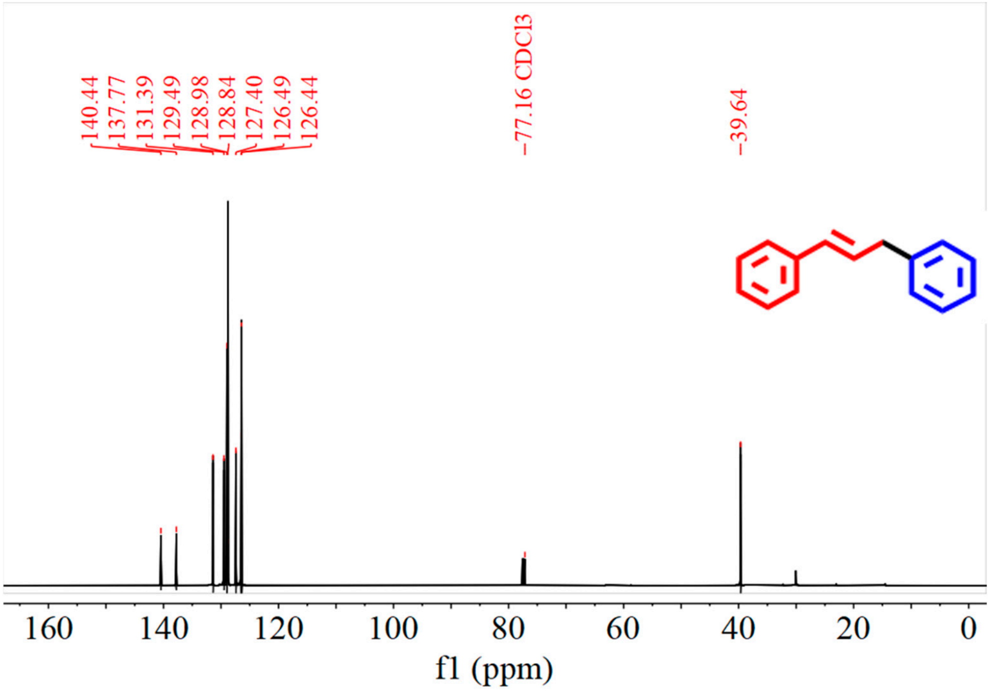

Figure S2 ^{13}C NMR (151 MHz, Chloroform-d) δ 140.44, 137.77, 131.39, 129.49, 128.98, 128.84, 127.40, 126.49, 126.44, 39.64

(2) Biphenyl: ^{1}H NMR and ^{13}C NMR spectrum (in $CDCl_3$):

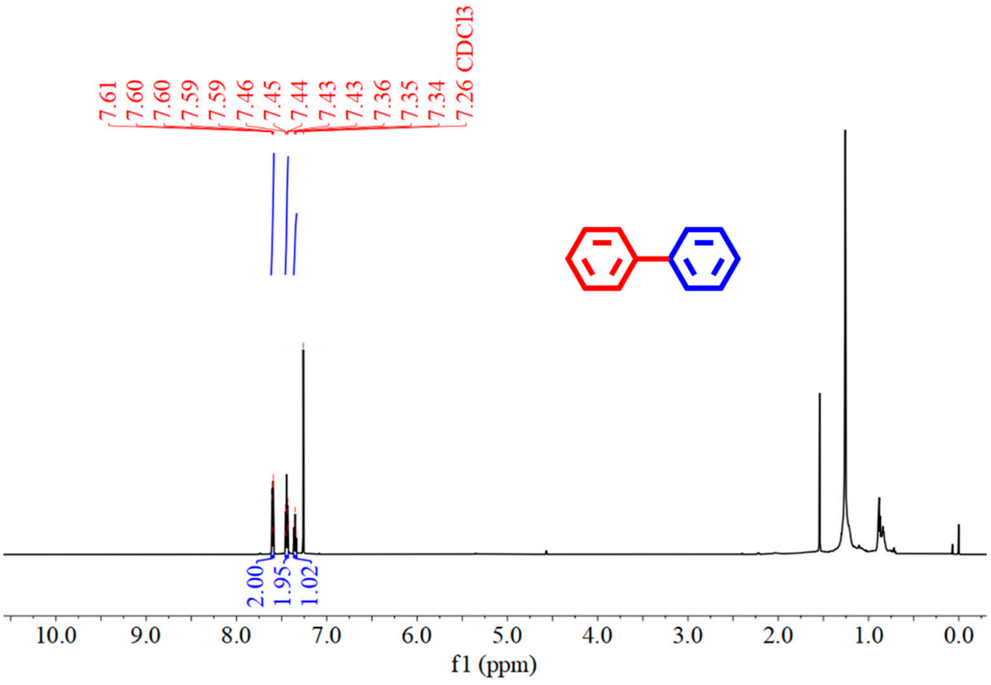

Figure S3 ^{1}H NMR (600 MHz, Chloroform-d) δ 7.62 – 7.58 (m, 2H), 7.44 (d, J = 15.4 Hz, 1H), 7.35 (t, J = 7.4 Hz, 1H)

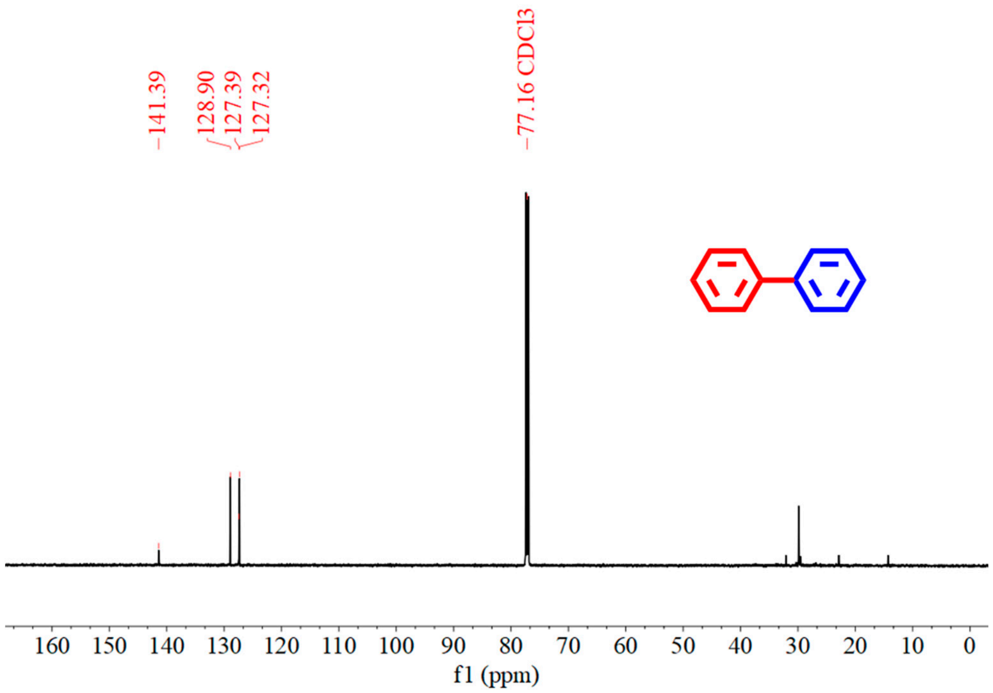

Figure S4 ^{13}C NMR (151 MHz, Chloroform-d) δ 141.39, 128.90, 127.39, 127.32

(3) 4-methylbiphenyl: ^{1}H NMR and ^{13}C NMR spectrum (in $CDCl_3$):

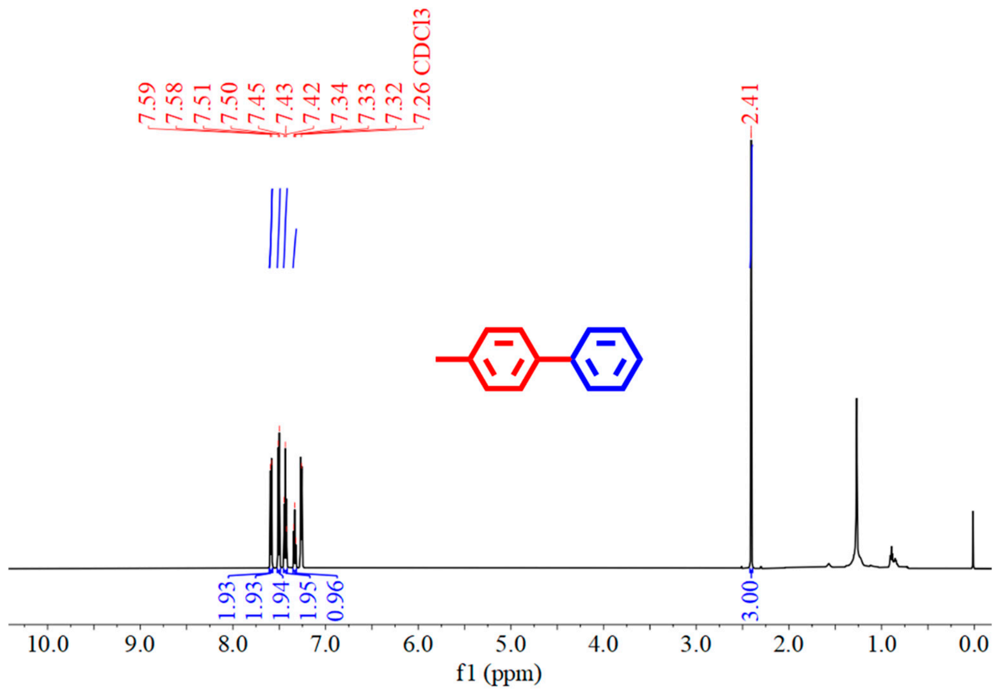

Figure S5 ^{1}H NMR (600 MHz, Chloroform-d) δ 7.59 (d, J = 8.1 Hz, 2H), 7.51 (d, J = 8.1 Hz, 2H), 7.44 (d, J = 7.7 Hz, 2H), 7.33 (t, J = 7.4 Hz, 1H), 2.41 (s, 3H)

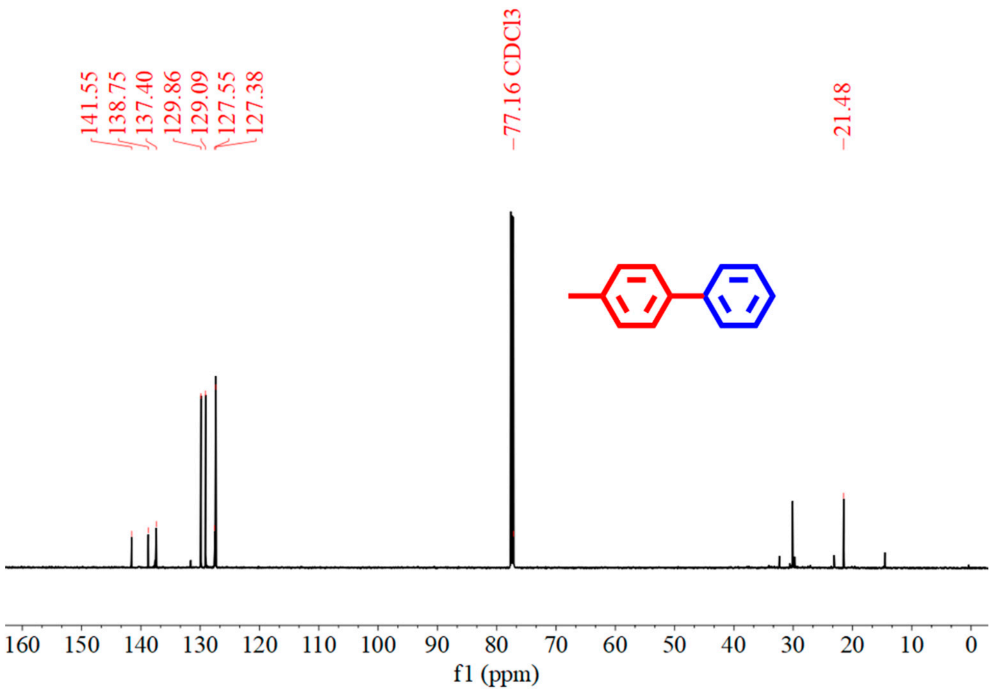

Figure S6 ^{13}C NMR (151 MHz, Chloroform-d) δ 141.55, 138.75, 137.40, 129.86, 129.09, 127.55, 127.38, 21.48

(4) 4-nitrobiphenyl: ^{1}H NMR and ^{13}C NMR spectrum (in $CDCl_3$):

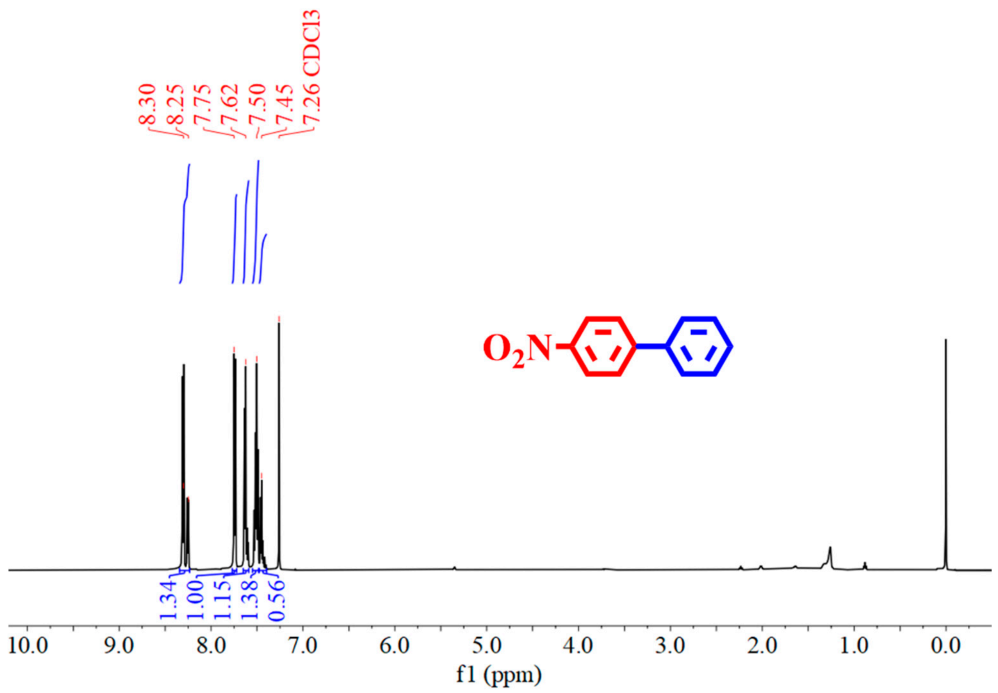

Figure S7 ^{1}H NMR (600 MHz, Chloroform-d) δ 8.25 (s, 0H), 7.75 (s, 2H), 7.62 (s, 2H), 7.50 (s, 3H)

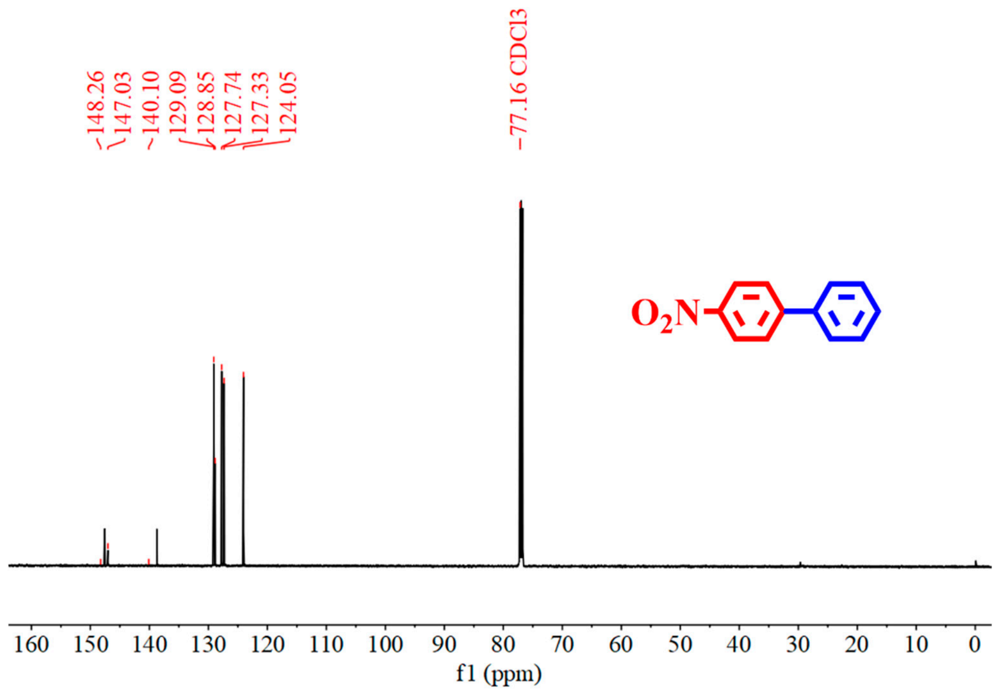

Figure S8 ^{13}C NMR (151 MHz, Chloroform-d) δ 147.03, 129.09, 128.85, 127.74, 127.33, 124.05
